# Yak whole-genome resequencing reveals domestication signatures and prehistoric population expansions

**DOI:** 10.1038/ncomms10283

**Published:** 2015-12-22

**Authors:** Qiang Qiu, Lizhong Wang, Kun Wang, Yongzhi Yang, Tao Ma, Zefu Wang, Xiao Zhang, Zhengqiang Ni, Fujiang Hou, Ruijun Long, Richard Abbott, Johannes Lenstra, Jianquan Liu

**Affiliations:** 1 State Key Laboratory of Grassland Agro-Ecosystem, College of Life Science, Lanzhou University, Lanzhou 730000, China; 2 MOE Key Laboratory for Bio-resources and Eco-environment, College of Life Science, Sichuan University, Chengdu 610064, China; 3 School of Biology, University of St Andrews, St Andrews, Fife KY16 9TH, UK; 4 Faculty of Veterinary Medicine, Utrecht University, Yalelaan 8, 3584 CM Utrecht, The Netherlands

## Abstract

Yak domestication represents an important episode in the early human occupation of the high-altitude Qinghai-Tibet Plateau (QTP). The precise timing of domestication is debated and little is known about the underlying genetic changes that occurred during the process. Here we investigate genome variation of wild and domestic yaks. We detect signals of selection in 209 genes of domestic yaks, several of which relate to behaviour and tameness. We date yak domestication to 7,300 years before present (yr BP), most likely by nomadic people, and an estimated sixfold increase in yak population size by 3,600 yr BP. These dates coincide with two early human population expansions on the QTP during the early-Neolithic age and the late-Holocene, respectively. Our findings add to an understanding of yak domestication and its importance in the early human occupation of the QTP.

## Abstract (web summary)

Based on whole genome resequencing of more than 80 wild and domestic yaks from high-altitude Qinghai-Tibet Plateau, this study recovers demographic and genetic processes underlying the domestication of this animal. Qiu *et al.* also identify genes and associated pathways as candidates for selection during the domestication process.

## Graphical abstract

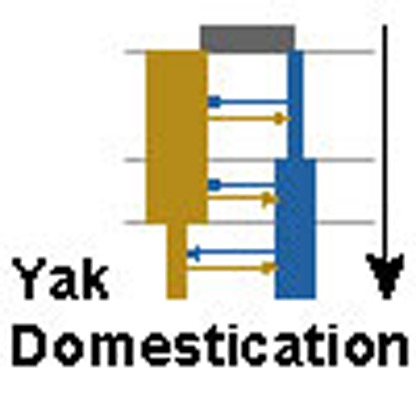

Domestication of livestock species was a key factor in triggering the socioeconomic transition in humans from a hunter–gatherer lifestyle to one of nomadic pastoralism or agricultural settlement^1^,^2^. This process occurred first in the Middle East ∼11,000 years ago and later in other parts of the world. The Qinghai-Tibet Plateau (QTP) is the world's largest and highest plateau with an area of ∼2.5 million square kilometres and an average elevation of 4,200 m. Humans are known to have colonized this vast area of Asia by at least 20,000 years ago and subsequent large-scale human population expansions occurred during the early Neolithic (10,000–7,000 years before present (yr BP)) and late Holocene (4,000–3,000 yr BP)^3^,^4^,^5^. The bovine yak species is endemic to this region. Historical records and archaeological evidence suggest that yak pastoralist societies were established in the QTP by ∼4,500 yr BP (refs 6, 7) and previous analyses of mitochondrial DNA variation indicate that yaks were domesticated during the early Neolithic period, some time between 6,000 and 12,000 yr BP (refs 8, 9). Since then, yak has become the mainstay of Tibetan pastoral society and >14 million domestic yaks are currently kept on the QTP, providing food, shelter, fuel and transport for the indigenous human population^10^,^11^. The ancestral wild yak population is threatened, but still exists with regular gene flow occurring between wild and domestic populations (Supplementary Note 1). Because yak domestication preceded the development of a human pastoral lifestyle in the QTP, a plausible hypothesis is that yak domestication was closely associated with early human population expansion in the region.

To examine the domestication of yak in more detail, we compare the genomes of wild and domestic yaks and investigate genetic changes underlying domestication. We use coalescent modelling to date yak domestication and population expansions more precisely than can be inferred from available archaeological and palaeontological evidence, and relate our findings to information on the prehistoric development of human society on the QTP.

## Results

### Genome resequencing and genetic variation

**Figure 1:**
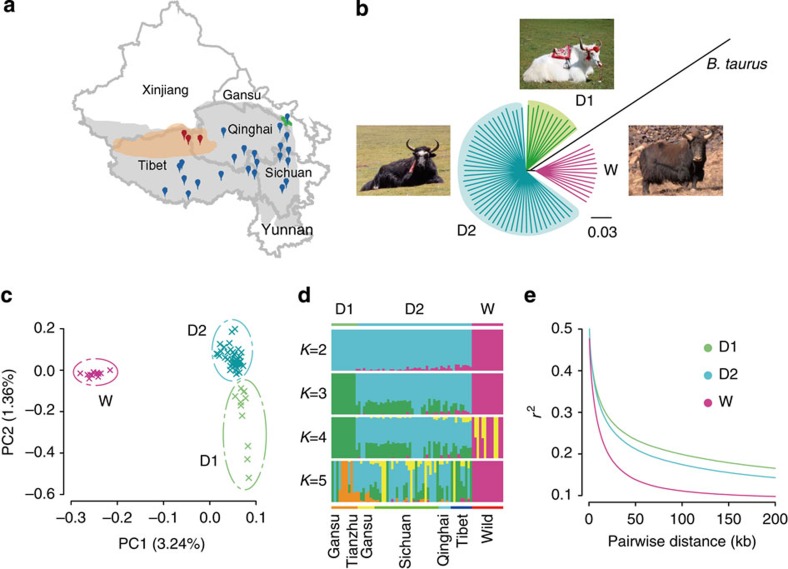
Phylogenetic and population genetic analyses of wild and domestic yaks. (**a**) The geographic distribution of the sampling locations for wild (dark red) and domestic (dark blue) yaks. The coloured areas indicate geographic distribution of wild yaks (light red), Tianzhu county (green) and the QTP (grey). (**b**) A neighbour-joining phylogenetic tree constructed using whole-genome SNPs data. The scale bar represents level of similarity. W: wild yaks; D1, the Tianzhu white breed; and D2: all of the remaining domestic yaks. (**c**) Principal component (PC) analysis plots of the first two components. The fraction of the variance explained is 3.24% for PC1 and 1.36% for PC2, with Tracy–Widom *P*<10^−44^ (Supplementary Table 3). (**d**) Population structure plots with *K*=2–5. The *y* axis quantifies the proportion of the individual's genome from inferred ancestral populations, and *x* axis shows the different populations. Geographic information is provided in Supplementary Table 1. (**e**) Decay of linkage disequilibrium of D1, D2 and W populations measured by *r*^2^.

We analysed genome sequences from 13 wild yaks, representing three highly diverged mitochondrial lineages^8^,^9^, and 59 domestic yaks from different locations on the QTP (Fig. 1a) representing 48 animals from unselected landraces (D2 population) as well as 11 Tianzhu white yaks (D1 population), which since 130 years ago^11^,^12^ have been bred by strict selection of coat colour (Fig. 1b). Genome resequencing accomplished an average depth of 6.7 × and average genome coverage of 98% (Supplementary Table 1). We detected a total of 14.56 million high-quality single nucleotide polymorphisms (SNPs), most of which (76.4%) are located in intergenic regions (Supplementary Table 2).

### Genetic changes underlying domestication

To examine the genome-wide relationships and divergence between wild and domestic yak populations, we visualized pairwise genetic distances in a neighbour-joining tree (Fig. 1b, consensus tree based on 1,000 bootstrap replicates shown in Supplementary Fig. 1). This revealed a clear split between wild and domestic yaks despite continuing gene flow between them, and also separation of Tianzhu white yaks within the domestic population (Supplementary Note 1). Principal component analysis as well as model-based clustering yielded similar results (Fig. 1c,d; Supplementary Note 1; and Supplementary Table 3). Domestication often reduces effective population size (*N*e) and genetic diversity^13^,^14^; but we obtained similar sequence diversity (*π*) values of 0.0013 and 0.0014 for wild and domestic yaks, respectively (Supplementary Fig. 2 and Supplementary Table 4). We further found that the estimated population-differentiation statistic (*F*_ST_) between wild and domestic yaks is only 0.058 (Supplementary Table 4), which is smaller than between taurine and zebu cattle or between diverged taurine cattle breeds^15^. *F*_ST_ estimates supported the gene flow occurring between wild and domestic yaks (Supplementary Note 1).

**Figure 2:**
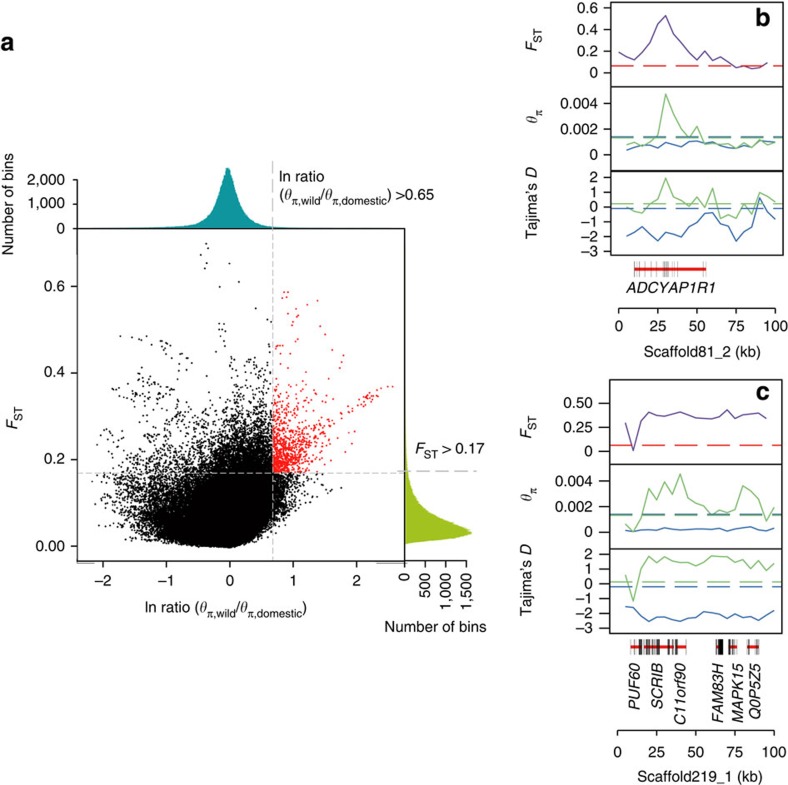
Genomic regions with selection sweep signals in domestic yaks. (**a**) Distribution of ln ratio (*θ*_*π*,wild_/*θ*_*π*,domestic_) and *F*_ST_ of 50 kb windows with 10 kb steps. Red dots represent windows fulfilling the selected regions requirement (corresponding to *Z* test *P*<0.005, where *F*_ST_≥0.17 and ln ratio≥0.65). Example of genes (**b**,**c**) with selection sweep signals in domestic yaks. *F*_ST_, *θπ* and Tajima's *D* values are plotted using a 5-kb sliding window. Wild (green) and domestic (blue) yaks are represented by different colours. Horizontal dashed lines represent mean whole-genome of corresponding values. Genes are shown at the bottom (black rectangle, coding sequences; red line, introns).

We detected genomic regions that have been subject to selection as inferred from high wild/domestic *π* log-ratios and an extreme divergence of allele frequencies of wild and domestic yaks^16^,^17^ (Fig. 2a and Supplementary Fig. 3). We identified 182 potential selective-sweep regions with an average size of 79.5 kb, together comprising around 14.5 Mb or 0.54% of the assembled genome. The role of these regions is confirmed by significantly lower values of Tajima's *D* and higher linkage disequilibrium patterns (*P* values 2.7 × 10^−12^ and 1.5 × 10^−4^, respectively, Wilcoxon rank-sum test, Supplementary Note 2) in domestic populations. These regions harbour 209 annotated protein-coding genes (Supplementary Data 1), which are expected to represent targets of selection. Among these, GO group GO:0051969 (regulation of transmission of nerve impulse) was overrepresented (*P*<0.05, Supplementary Table 5 and Supplementary Data 1) with eight genes affecting synaptic circuitry and neurological processes (*Arc, ASPA, ATP2B2, MYO6, NTRK2, Rab40c, SNCA* and *TG*). From these genes and 30 other genes (Supplementary Data 1) involved in brain and neuronal development, 19 are considered to be associated with behaviour. *ADCYAP1R1* (Fig. 2b) encodes a pituitary adenylate cyclase-activating polypeptide receptor that in humans is strongly expressed in the amygdala and hippocampus, and is associated with fear response, threat stimuli, post-traumatic stress disorder and other anxiety disorders. *Adcyap1r1*-deficient mice exhibit strongly reduced anxiety-like behaviour^18^. *SCRIB* (Fig. 2c) encodes the scribbled planar cell polarity protein, which is a key regulator of brain development and spine morphology. *Scrib1* knockout mice exhibit enhanced learning and memory abilities and impaired social behaviour correlated with altered neuronal morphology^19^. *PLXNB1* encodes a neuronal receptor for semaphorins and has an important role in developing nervous systems and controlling axon guidance^20^. A recent quantitative trait loci study in rat identified *PLXNB1* as a candidate gene contributing to differences in tameness and aggression^21^, which are expected to be important during the early phase of animal domestication^2^. The pathways of brain and neuronal development identified here to be under selection during yak domestication are similar to those reported previously for rabbit^22^ and cat^23^, suggesting common features of domestication in these unrelated species.

Only a few genes subject to selection were associated with specific physical characteristics or economically significant traits, such as *TTLL1* and *RHPN1* associated with sperm development and *RHOD* with early pregnancy. Also, a limited number of sweeps associated with coat colour were detected from an examination of genetic divergence between Tianzhu white yaks and other domestic yaks (Supplementary Note 3). In line with the low level of genetic and morphological differentiation recorded between wild and domestic yaks (Supplementary Table 4 and Supplementary Fig. 4), our analyses confirm that the effects of domestication in yaks are not as marked as for most other domestic species^1^,^22^. This may reflect a trade-off between survival of yaks in a harsh high-altitude environment and performance under pastoral conditions.

### Demographic history

**Figure 3:**
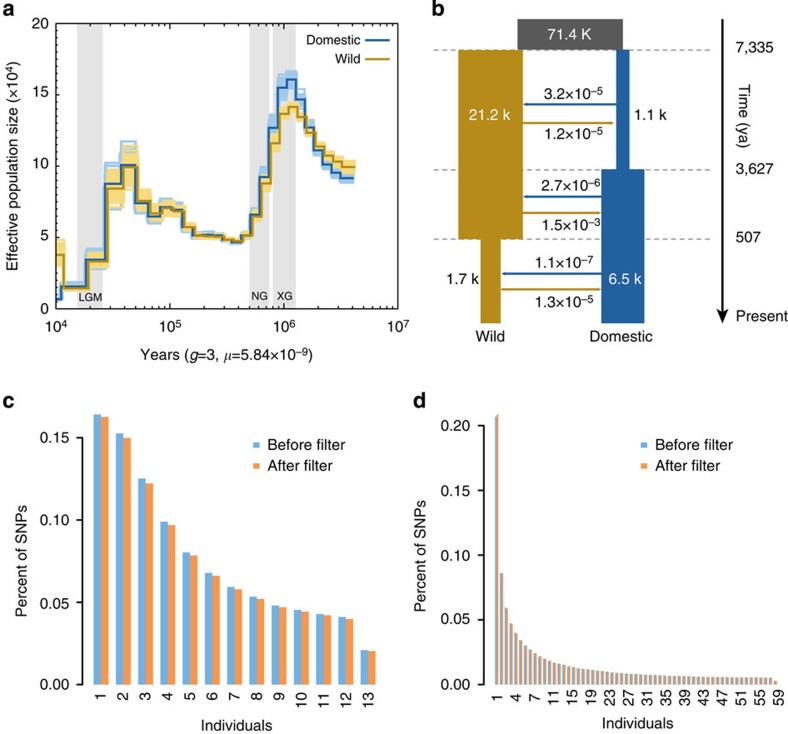
Demographic history of yak. (**a**) Demographic history inferred by PSMC. The period of the Xixiabangma Glaciation (XG, 1,170–800 thousand years ago, kya), Naynayxungla Glaciation (NG, 780–500 kya) and the last glacial maximum (LGM, ∼20 kya) are shaded in grey. (**b**) Schematic of demographic scenario modelled in Fastsimcoal2. The ancestral population is in grey, wild yak in brown and domestic yak in blue. The width shows the relative effective population size. The figures at the arrows indicate the average number of migrants per generation between wild and domestic yaks. The folded genome-wide SFS from 13 wild yaks (**c**) and 59 domestic yaks (**d**). Different colours represent data before (blue) and after (orange) impute filtering of sites for which the correlation of observed and imputed date was <0.9.

We employed the pairwise sequentially Markovian coalescent (PSMC) method^24^ to examine changes in effective population size (*N*_e_) of the ancestral population of both wild and domestic yaks in response to Quaternary climatic change. We applied this method to our deep-coverage (>20 ×) yak genomes from three wild and four domestic yaks, including the reference genome. Both wild and domestic yaks exhibited similar demographic trajectories until about 20,000 years ago (Fig. 3a and Supplementary Fig. 5). The ancestral *N*_e_ of yaks shows a peak at ∼1 Myr ago followed by two distinct declines. The first decline occurred ∼0.9 Myr ago, coinciding with extensive glaciation during the mid-Pleistocene^25^, with three highly divergent mitochondrial lineages known to have survived this decline^9^. Other animal species such as giant panda and golden snub-nosed monkeys living in the southern and southeastern QTP also suffered during the same period decreases in effective population size^26^,^27^. The second decline involved at least a threefold decrease in *N*_e_, and occurred ∼40,000 years ago coinciding with the last glaciation^25^.

We used the joint site frequency spectrum (SFS) approach implemented in fastsimcoal2 (ref. 28) to simulate more recent demographic fluctuations. Thirty alternative models of historical divergence were fitted to the allele-frequency spectrum of domestic and wild yak populations, incorporating strict isolation, isolation-with-migration, bottlenecks and/or growth (Supplementary Fig. 6). A demographic model in which domestic and wild yaks diverged through a dynamic process involving population bottlenecks in both wild and domestic yaks and extensive post-domestication gene flow produced a significantly better fit than alternative models (Fig. 3b). The allele-frequency spectrum simulated with the best model was very close to the spectrum generated from real data (Supplementary Fig. 7), demonstrating the accuracy of the calculations. Thus in the best fitting model domestication of yaks occurred ∼7,300 yr BP, with a 95% confidence interval of 7,227–7,914 yr BP, slightly later than the domestication of many other livestock species (10,000–8,000 yr BP), but preceding the introduction of taurine cattle to China 5,400–4,700 yr BP (ref. 29).

Analyses of mitochondrial, Y-chromosomal and autosomal DNA data suggest that modern humans began colonizing the QTP ∼30,000 yr BP and that their population size expanded rapidly first between 10,000 and 7,000 yr BP and later between 4,000 and 3,000 yr BP (refs 5, 30, 31). However, archaeological and anthropological evidence indicates that the earliest agricultural settlements in the northeastern QTP were established 5,200 yr BP or later^3^,^32^. During the early-Neolithic age, the climate on the QTP was warmer than today^25^, which may have favored persistence of a hunter–gatherer population in the region. Our results suggest that the yak was domesticated by 7,300 yr BP and may have been triggered by and facilitated the first expansion of human population size on the QTP at this stage. Given the absence of agricultural settlements at this time, the first pastoralists were probably nomadic herders. A similar domestication by nomadic people in another extreme environment has been described for reindeer^33^.

Later in the Holocene agriculture was established on the QTP, for example, the introduction of barley cultivation 4,000–3,600 yr BP (ref. 30). This coincided with a second human population expansion^3^,^4^,^5^,^30^,^32^ despite the colder climate of the late Holocene^25^. Interestingly, our coalescence analyses revealed a sixfold increase in population size of the domestic yak (*N*_e_, from 1,100 to 6,500) during the same period (3,600 yr BP, Fig. 3b), which might have resulted from the second human population expansion on the QTP following the introduction of agriculture or contributed to this second expansion by providing a reliable resource of food, hides and transportation. According to our coalescent analysis, ∼500 years ago the *N*e of the wild yak population seriously declined from 21,200 to 1,700, which is consistent with a loss of low-frequency variants (Fig. 3c,d) and a lower genetic diversity in current wild yaks (Supplementary Fig. 2 and Supplementary Table 4). This possibly resulted from habitat loss due to increasing human activities.

## Discussion

Despite low morphological divergence and continuing gene flow, we detected a clear genetic split between wild and domestic yaks. We found that the genomes of domestic yaks exhibit clear signatures of selection at genes that probably affect animal behaviour and tameness according to previous reports on other animals^22^,^23^. These findings suggest that parallel processes of evolution have occurred during the domestication of unrelated animals across different localities of the world. Our study further indicates that the yak is likely to have been domesticated before 7,000 years ago and that domestication was closely associated with the expansion of the human population on the QTP during the early Neolithic period^31^. Moreover, following the introduction of agriculture^30^, a further increase in the effective population size of domestic yaks later in the Holocene may have resulted from or contributed to causing a second human population expansion and the subsequent development of human society on the QTP during this period^3^,^4^,^5^,^30^,^32^

## Methods

### Sample collection and sequencing

A total of 84 individuals (15 wild yaks and 69 domestic yaks, Supplementary Table 1) were collected and sequenced, yielding a data set of genomes from 13 wild and 59 domestic yaks without close relatives and with <50% missing data. The wild samples were collected from corpses of wild yaks in the central Kokohili region, which were identified as wild yaks because of their long hair and large skeletons. Domestic yaks were sampled across the species main geographic distribution. Samples were collected under the supervision of ethical committees and permission was obtained when necessary. For each yak, genomic DNA was extracted from muscle samples using a standard phenol/chloroform extraction^34^. The quality and integrity of the extracted DNA was checked by measuring the A260/A280 ratio and by agarose gel electrophoresis. Paired-end sequencing libraries with an insert size of 500 bp were constructed according to the Illumina manufacturer's instructions for sequencing on the Hiseq 2,000 platform. Sequencing and base calling were performed according to the standard Illumina protocols.

### Sequence quality checking

Duplicate reads caused by base-calling and adaptor contamination were removed. Reads with (i) ≥10% unidentified nucleotides (*N*), (ii) with a phred quality ≤7 for >65% of the read length or with (iii) a stretch of >10 bp identical to the adaptor sequence with up to two mismatches were removed or corrected using a k-mer frequency-based methodology^35^. Reads were also trimmed if they had three consecutive bp with a phred quality of ≤13, and discarded if they were shorter than 45 bp.

### Sex-linked scaffolds

We used Blastz (ref. 36) to perform whole-genome alignment of the yak and taurine cattle genomes and to identify yak sex chromosomes (downloaded from National Center for Biotechnology Information, http://www.ncbi.nlm.nih.gov, UMD3.1.1, GCA_000003055.4). All hits against the cattle sex chromosome were treated as sex-linked scaffolds. A total of 186 scaffolds with a combined size of ∼134 Mb were aligned to the cattle sex chromosome and omitted from subsequent analyses.

### Read mapping

High-quality reads were aligned to the *Bos grunniens* reference genome^37^ and mitochondrial reference genome (accession number: JQ692071.1) using BWA-MEM (0.7.10-r789) with default parameters^38^. Sequence Alignment/Map (SAM) format files were imported to SAMtools (v0.1.19)^39^ for sorting and merging and *Picard* (http://broadinstitute.github.io/picard/, version 1.92) to assign read group information containing library, lane and sample identity. The Genome Analysis Toolkit (GATK, version 2.6–4-g3e5ff60)^40^ was used to perform local realignment of reads to enhance the alignments in the vicinity of indel polymorphisms. Realignment was performed with GATK in two steps. The first step used the RealignerTargetCreator to identify regions where realignment was needed, and the second step used IndelRealigner to realign the regions found in the first step, generating for each individual a realigned Binary sequence Alignment/Map (BAM) file.

### Filtering alignments

We removed all alignments that were not of sufficiently high quality for SNP detection and subsequent analyses. Alignments to be removed were identified using the following stepwise protocol: (i) discard reads that do not map uniquely; (ii) only use reads for which a mate can be mapped; (iii) discard ‘bad' reads with flag ≥255; (iv) discard bases with a quality <20; and (v) discard reads with a mapping quality <30. We also adjusted the quality scores around indels using SAMtools and removed the alignments that anchored short scaffolds of <2 kb.

### Filtering sites

To minimize the influence of sequencing and mapping bias, the following site types were discarded: (i) sites with unbalanced quality scores as determined using Wilcoxon rank-sum test with threshold of *P*<10^−5^; (ii) sites with strand bias (*P*<10^−5^); (iii) sites with extremely low (<2 ×) or extremely high (>18 ×) coverage, both thresholds being defined after investigating the coverage distribution empirically; (iv) sites that failed the Hardy–Weinberg Equilibrium test and *P*<10^−3^, using SAMtools and BCFtools^39^; and (v) sites for which the available information derived from <90% of the sampled domestic and/or wild populations. The combined application of these filters left us with a data set comprising ∼2.2 Gb, representing 81.7% of the genome.

### SNP and genotype calling

Variant discovery analysis was conducted at the population level for wild and domestic yak samples separately. We used the SAMtools model^41^ implemented in analysis of next generation sequencing data (ANGSD)^42^ to estimate genotype likelihoods and generated Beagle files. A maximum likelihood approach^43^ was then used to infer major/minor states based on the genotype likelihoods. Minor allele-frequency polarized by major/minor state was also estimated from the genotype likelihoods based on Kim's method^44^. A likelihood ratio test statistic for the allele-frequency based on a *χ*^2^ distribution with one degree of freedom and a *P*-value threshold of 1 × 10^-6^ was used as an SNP discovery criterion. SNPs were retained only if they could be genotyped in at least 90% of the sampled individuals from both domestic and wild populations. This yielded a total of 14.6 million SNPs.

A two-step procedure implemented in ANGSD was used to estimate the SFS: (i) sample allele-frequency likelihood files (.saf) were generated using the option ‘–doSaf 1', with ancestral state being assigned by a cattle genome^45^ (17.4 ×); (ii) the allele-frequency likelihood files were optimized using the realSFS (ref. 46) programme in order the estimate the SFS. Genotypes were called using the full set of genotype likelihoods data. Using the sample allele frequency as a prior for genotype frequencies under the assumption of Hardy–Weinberg equilibrium, we then computed the posterior probabilities of the genotypes at each site for each individual.

### Relationships

To identify closely related individuals, the programme PLINK v1.07 (ref. 47) was used to obtain pairwise estimates of Identity-By-State (IBS) scores between all samples. One wild and seven domestic individuals were excluded due to their high pairwise genetic similarity with another sampled individual (IBS>0.9), leaving only unrelated samples for use in the downstream analyses. We also discarded one wild and three domestic individuals with >50% missing data (Supplementary Table 1).

### Genome-wide identity scores

To visualize genetic relatedness between domestic and wild populations, we calculated for individual SNPs identity scores as the sum of the products of the frequencies of both alleles with the frequencies of the same allele in the reference genome. Identity scores for 50 kb windows along the genome were averaged over the SNPs within the window (Supplementary Fig. 4).

### Population genetics analysis

After mapping sequencing data against the reference yak mitochondrial genome (accession number: JQ692071.1), only positions covered by a minimum number of three independent unique reads with base qualities of ≥30 were used to call the consensus sequences. Eighty yak mitochondrial sequences were generated and aligned together with 81 sequences from *B. grunniens*, one from *Bos taurus*, one from *Bos indicus*, one from *Bison bison* and one from *Bison bonasus* (see the labels of external branches on Supplementary Fig. 8 for accession numbers). We partitioned the alignment into six main regions: the D-loop, ribosomal RNA, tRNA and the first, second and third codon positions for Coding DNA Sequence (CDS). The initiation and termination codons and overlapping regions between CDSs were excluded. We also removed sites with missing genotypes in >10% of the sampled individuals. The best mutational model for each of the partitions was then selected using ModelGenerator v851 (ref. 48) with eight rate categories. The partitions and their corresponding mutational models were used for Bayesian phylogenetic inference with MrBayes v3.22 (ref. 49), running two analyses in parallel, each with four Markov Chain Monte Carlo (MCMC) chains. The final tree topology was recovered after a total of 100,000,000 generations, sampling 1 in every 1,000 generations after discarding the first 25% as burn-in. The s.d. of split frequencies was below 0.01 after 100,000,000 generations, indicating the convergence of the four chains to the stationary distribution. The resulting tree, as drawn with MEGA v5.0 (ref. 50), is shown in Supplementary Fig. 1. This tree topology is consistent with the presence of distinct phylogeographical patterns and multiple divergent lineages in yaks as determined in previous studies^8^,^9^ based on D-loop and mitochondrial genomic sequences from a wide range of samples.

For autosomal genome data, a neighbour-joining tree was constructed with PHYLIP v3.695 (http://evolution.genetics.washington.edu/phylip.html) using the matrix of pairwise genetic distances. The ancestral states of the SNPs were determined using the close relative of the yak, *B. taurus*^45^, as the outgroup. A second frequency tree (Supplementary Fig. 1) was generated based on 1,000 bootstrap replicates using the consensus module of PHYLIP. FigTree (http://tree.bio.ed.ac.uk/software/figtree/) and MEGA v5.0 were used to visualize the phylogenetic trees. Principal component analysis of the SNPs was performed using the smartpca programme in EIGENSOFT v5.0 (ref. 51). A Tracy–Widom test was used to determine the significance level of the eigenvectors.

Geographic distances in km between individuals were calculated via the formula: Distance=*a*cos(sin(lat1 × *π*/180)sin(lat2 × *π*/180)+cos(lat1 × *π*/180)cos(lat2 × *π*/180)cos(lon2 × *π*/180−lon1 × *π*/180)) × 6378.135, in which lat1 and lat2 are latitudes in degrees of the two individuals and lon1 and lon2 their longitudes (Supplementary Fig. 9).

### Genome-wide patterns of heterozygosity and neutrality tests

The nucleotide diversity (*π*), population-differentiation statistic (*F*_ST_), Tajima's *D* statistic and Watterson estimator (*θ*_w_) were calculated using a sliding window approach (50 kb window sliding in 10 kb steps)^52^,^53^,^54^. To compensate for missing data and variations in the depth-of-coverage across the different genomes (4.5–8.8 × , average coverage 6.7 ×), an empirical Bayesian method was used to calculate the posterior probabilities for the sample frequency spectrum using a maximum likelihood estimate of the SFS as the prior. The method takes genotype uncertainty into account and is based directly on genotype likelihoods rather than called genotypes. Only genomic windows in which at least 80% of bases were covered were considered to avoid coverage-related bias, leaving 207,111 windows with an average SNPs number of 346 per-window (min 34, max 3,298).

### Screening for selective sweeps

To identify genomic regions that may have been subject to selection during domestication, we combined the two domestic yak populations (D1 and D2) as a single domestic gene pool. We scanned the genome for regions with the highest differences in genetic diversity (*π* log-ratio wild/domestic) and extreme divergence in allele frequency between wild and domestic populations using a genome-wide sliding window strategy. More specifically, we calculated the sequence diversity statistics (*π*), and the population-differentiation statistic (*F*_ST_) using a 50 kb window with a 10-kb step. The *π* log-ratio was calculated as ln(*π*_W_)−ln(*π*_D_), where *π*_W_ and *π*_D_ are the nucleotide diversity values for the wild and domestic yaks, respectively. At a significance level of *P*<0.005 (*Z* test, with *π* log-ratio >0.65 and *F*_ST_>0.17, Fig. 2a and Supplementary Fig. 3), we identified a total of 182 potential selective-sweep regions (with an average size of 79.5 kb, range from 10 to 450 kb) overlapping with 209 candidate genes, used for subsequent analysis and discussion.

To test whether the candidate selective-sweep regions had an excess of singleton polymorphisms, we computed the Tajima's *D* value for domestic yaks using the same sliding window approach. Regions under selective sweeps had very significantly lower values of Tajima's *D* (*P*=2.7 × 10^−12^, Wilcoxon rank-sum test). In addition, pairwise *r*^2^ values showed that the candidate regions exhibited significantly extended linkage disequilibrium (*P*=1.5 × 10^−4^, Wilcoxon rank-sum test). These results confirm the occurrence of selective sweeps in the identified regions.

The impact of population structure to selection signal was tested by repeating the sweep detection by comparing the two domestic populations D1 and D2 separately to the wild population (W). For 196 (93.8%) of 209 genes selection signals were statistically significant in both domestic populations; relatively strong selection signals were evident for the other 13 candidate genes but did not reach the significance threshold (Supplementary Data 1).

Functional classification of GO categories was performed using the Blast2GO programme^55^. Enrichment analysis was performed and the *χ*^2^ test was used to calculate the statistical significance of enrichment. The *P* values were adjusted by FDR and the adjusted *P* value cut-off was 0.05.

### Demographic history

We inferred a demographic history for *B. grunniens* by applying the Pairwise Sequentially Markovian Coalescence model^24^ to the complete diploid genome sequences, excluding sexual chromosomes/scaffolds. This method reconstructs the history of changes in population size over time using the distribution of the most recent common ancestor (tMRCA) between two alleles in an individual. PSMC has high false-negative rates at low depth, which leads to a systematic underestimation of true event times. To ensure the quality of consensus sequences, we sequenced three wild and three domestic yaks to a high coverage of 20 × . DNA was prepared and libraries were built using the protocols described above. Consensus sequences were obtained using SAMtools and divided into non-overlapping 100 bp bins. Bases of low sequencing depth (less than a third of the average depth) or high depth (twice the average depth) were masked. The analysis was performed using the following parameters: −N25 −t15 −r5 −p ‘4+25 × 2+4+6'. The mutation rate per generation per site was estimated as: *μ*=*D* × *g*/2 *T* where *D* is the observed frequency of pairwise differences between two species, *T* is the estimated divergence time and g is the estimated generation time for the two species. The estimated generation time (*g*) was set to 3 years and the estimated divergence time was set to 4.7 Myr based on a previous study on cattle and yak^56^. These values yielded an estimated mutation rate of 5.84 × 10^−9^ mutations per generation per site for the yak. PSMC modelling was done using a bootstrapping approach, with sampling performed 100 times to estimate the variance of the simulated results.

As PSMC inference is known to be inaccurate for recent datings, we also inferred the joint demographic histories of the wild and domestic yak using the flexible and robust simulation-based composite-likelihood approach implemented in the fastsimcoal2 programme^28^, which infers demographic parameters from the SFS. The analysis was performed for 13 wild samples and 59 domestic samples. To improve the genotype accuracy and infer missing genotypes, we used BEAGLE (ref. 57) to infer the haplotypes of wild and domestic individuals from previously estimated genotypes. After investigating the empirical minor allele frequency distributions, we inferred haplotypes for non-coding sites alone with estimated minor allele frequency values of >0.038 for wild yak and >0.008 for domestic yak. Only sites for which the correlation between the observed and imputed data (*r*^2^) was >0.9 were retained. To examine potential bias introduced by impute filtering, we compared the SFS before and after filtering. No potential bias was found (Fig. 3c,d). The joint SFS of wild and domestic yaks was used to estimate evolutionary scenario parameters. We used the folded spectrum to minimize potential biases when determining the ancestral allelic states. Alternative models of historical events were fitted to the joint SFS of wild and domestic yak and we allowed only instantaneous population size changes (Supplementary Fig. 6). For each model, we ran the programme 50 times with varying starting points to ensure convergence, and retained the fitting with the highest likelihood. Estimates were obtained from 100,000 simulations per likelihood estimation (-n100,000, -N100,000), 40 Expectation/Conditional Maximization (ECM) cycle (-L40) and 50 runs per data set. The best model was addressed through the maximum value of the likelihoods and Akaike information criterion^28^. Parametric bootstrap estimates were obtained by parameter estimation based on 100 data sets simulated according to CML estimates in best model (model15) estimation parameters (Supplementary Data 2). The population history and parameters from the best model were used to perform forward simulation and residuals analysis with ∂a∂i (ref. 58) to check the accuracy of the demographic model.

## Additional information

**Accession codes:** The sequencing data for this project have been deposited in the European Nucleotide Archive (EMBL-EBI) under accession code PRJNA285834.

**How to cite this article:** Qiu, Q. *et al.* Yak whole-genome resequencing reveals domestication signatures and prehistoric population expansions. *Nat. Commun.* 6:10283 doi: 10.1038/ncomms10283 (2015).

## Supplementary Material

Supplementary Information Supplementary Figures 1-13, Supplementary Tables 1-6, Supplementary Notes 1-3 and Supplementary References

Supplementary Information Putative regions identified to be under domestication sweeps

Supplementary Information Parameters of models estimated in Fastsimcoal2

Supplementary Information Summary statistics of ABBA statistics
