# Supplementary material for: Yak whole-genome resequencing reveals domestication signatures and prehistoric population expansions
Source: Nat Commun. 2015 Dec 22;6:10283. doi: 10.1038/ncomms10283 (PMC4703879; doi:10.1038/ncomms10283)
Supplement: Supplementary Information — Supplementary Figures 1-13, Supplementary Tables 1-6, Supplementary Notes 1-3 and Supplementary References [file ncomms10283-s1.pdf]

Supplementary Figures

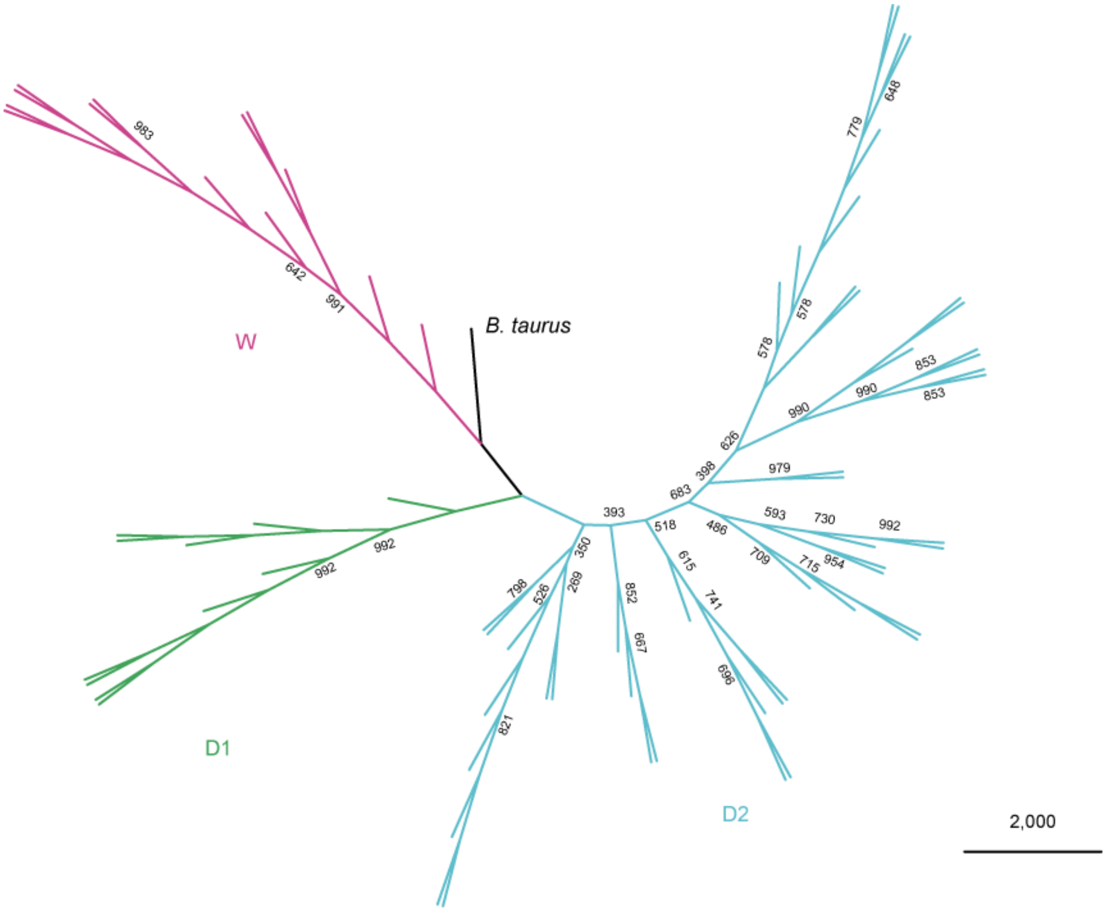

Supplementary Figure 1. Frequency tree based on 1000 boostrap replicates of autosomal genome data. Frequency tree generated based on 1000 bootstrap replicates. Bootstrap values less than 1,000 are shown on arcs; those equal to 1,000 are not shown. Different populations are labeled with different colors (W, red; D1, green; and D2, light blue).

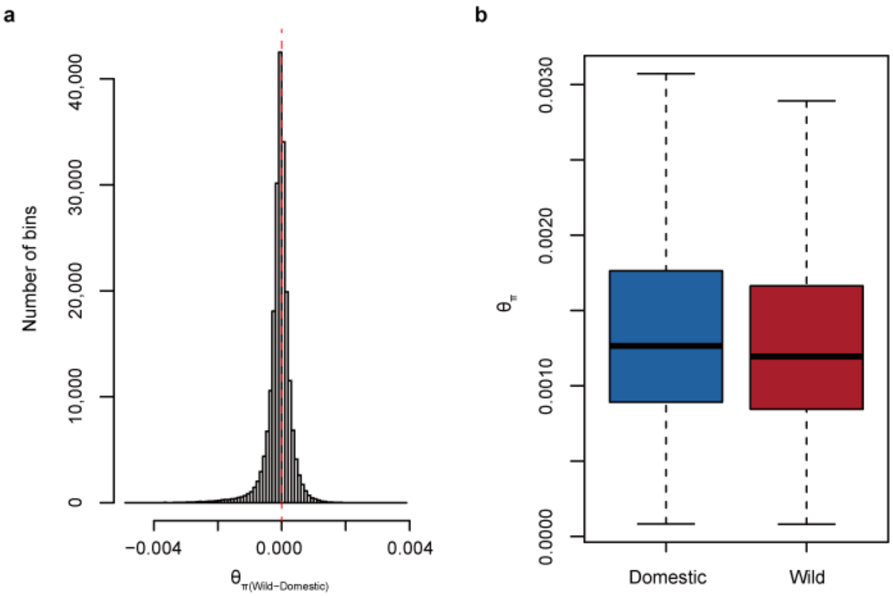

Supplementary Figure 2. Comparison of genome diversity between wild and domestic yaks. a, Distribution of difference of θ_π in 50 kb windows with 10 kb steps between wild and domestic yaks. **b**, Box plot of θ_π of wild and domestic yaks.

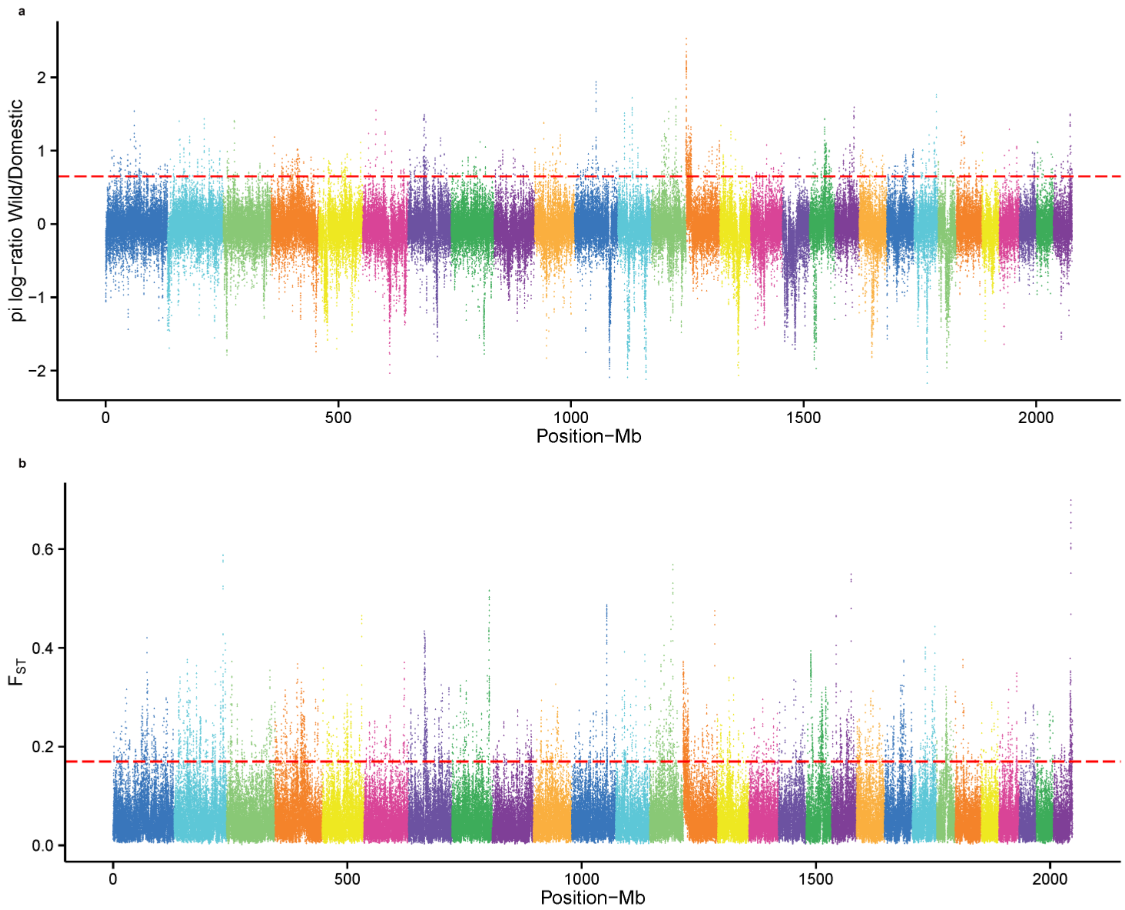

Supplementary Figure 3. Genome-wild distribution of ln ratio ($\theta_{\pi,wild}/\theta_{\pi,domestic}$) (a) and F_{ST} (b) between wild and domestic yaks. The horizontal dashed line indicates the threshold defining the selective sweeps (ln ratio ≥0.65 and F_{ST} ≥0.17).

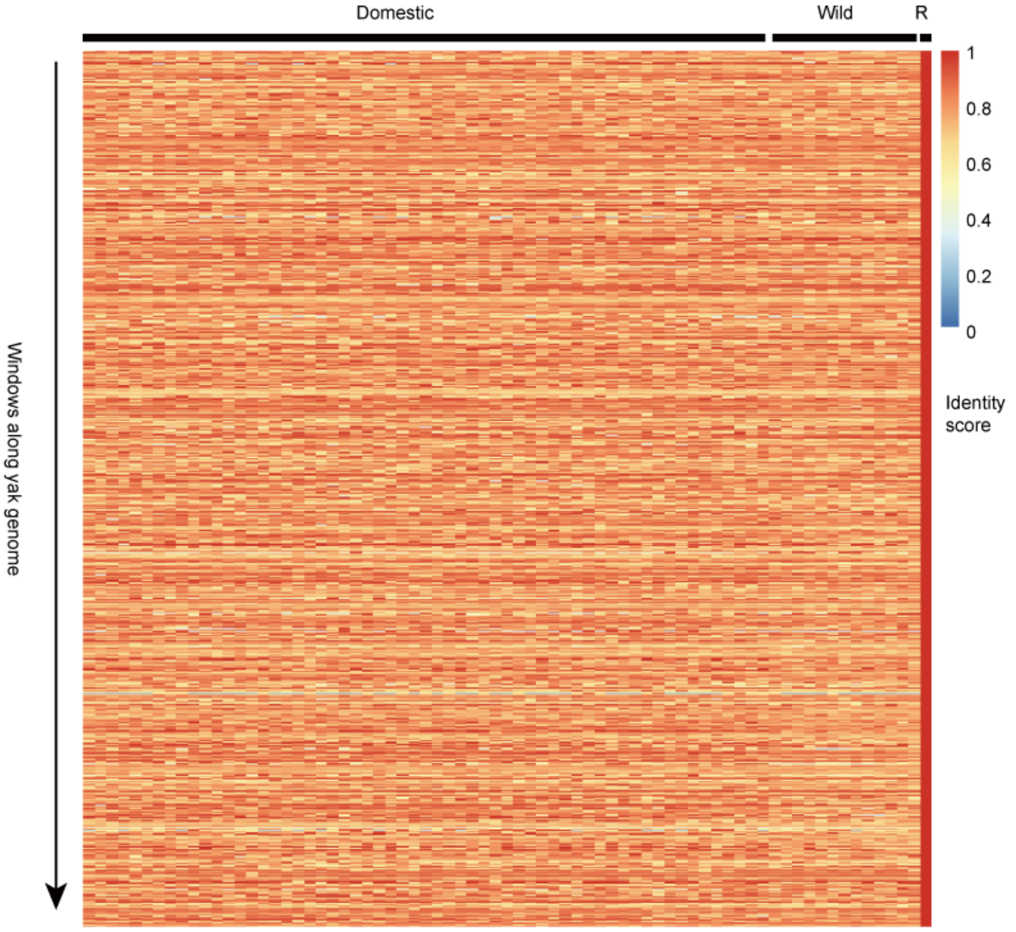

Supplementary Figure 4. Genetic relatedness between domestic and wild populations sampled in this study. Heat map (color code to the right) of identity scores based on comparing resequencing data with the assembly. The y-axis represents genome coordinates with chromosome 1 to 29 from the top to the bottom; the relative positions of scaffolds in chromosomes were identified by whole-genome alignment between yak and taurine cattle. The rightmost row (R) on the right represents the reference yak against itself.

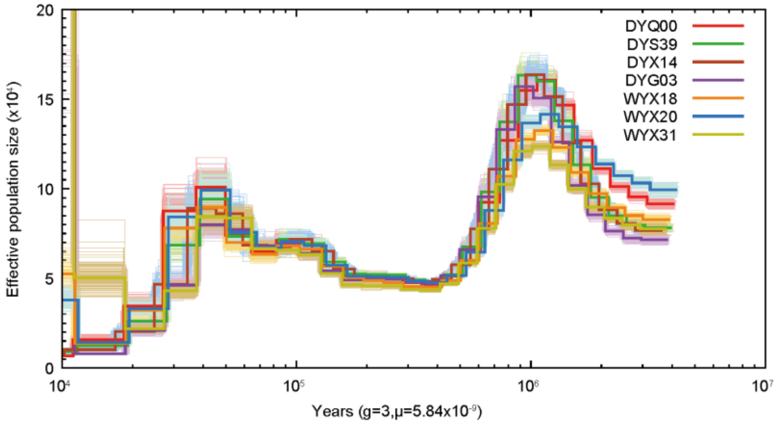

Supplementary Figure 5. Population history. Inferred historical population sizes by pairwise sequential Markovian coalescence analysis from autosomes sequences of three wild and four domestic individuals. g: generation time in years, μ: mutation rate per base per generation.

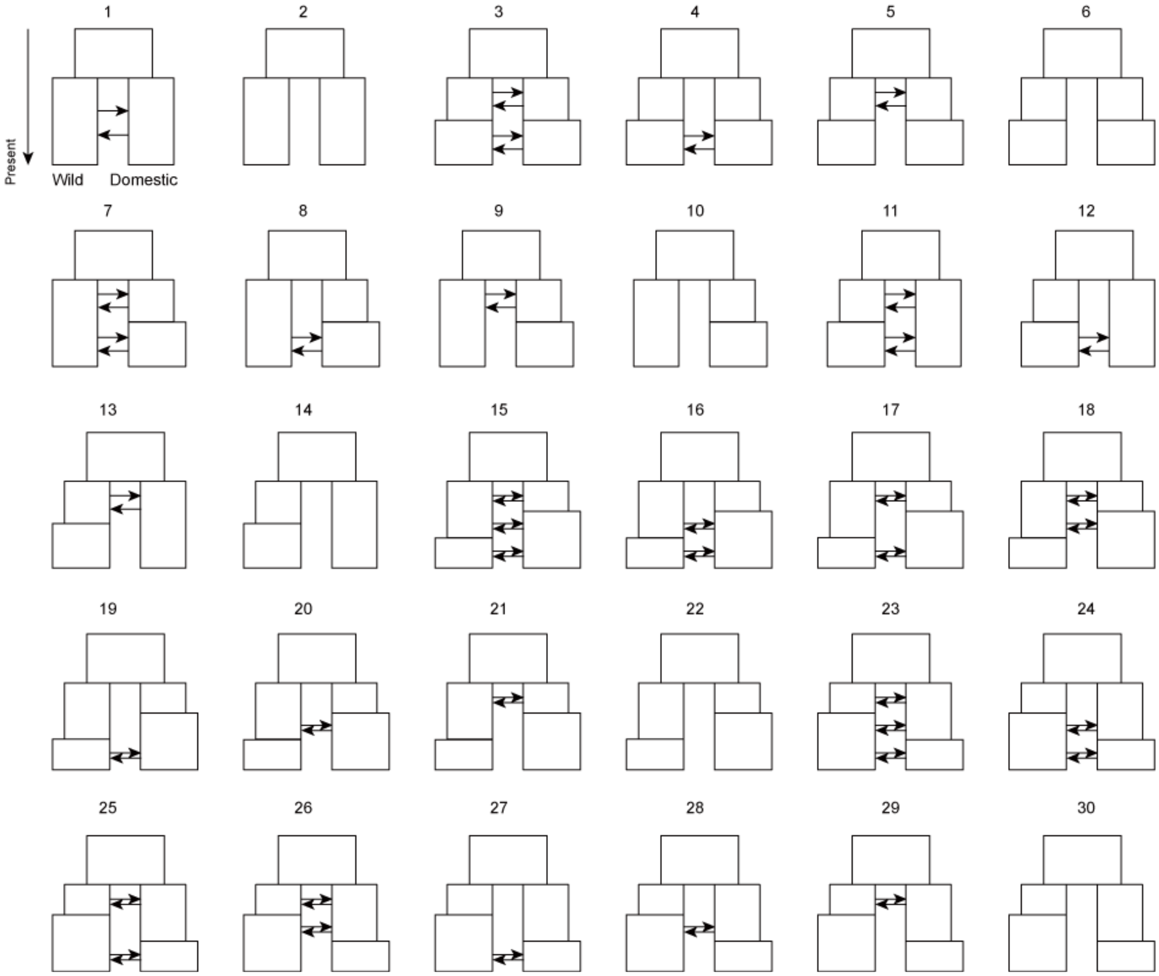

Supplementary Figure 6. Schematic diagram of different models of yak demographic history from ancient to recent estimated in Fastsimcoal2. The width of the box indicates population sizes of wild (left) and domestic (right) yaks. Arrows show the migrants between two populations. For detailed parameters, see Supplementary Data 2).

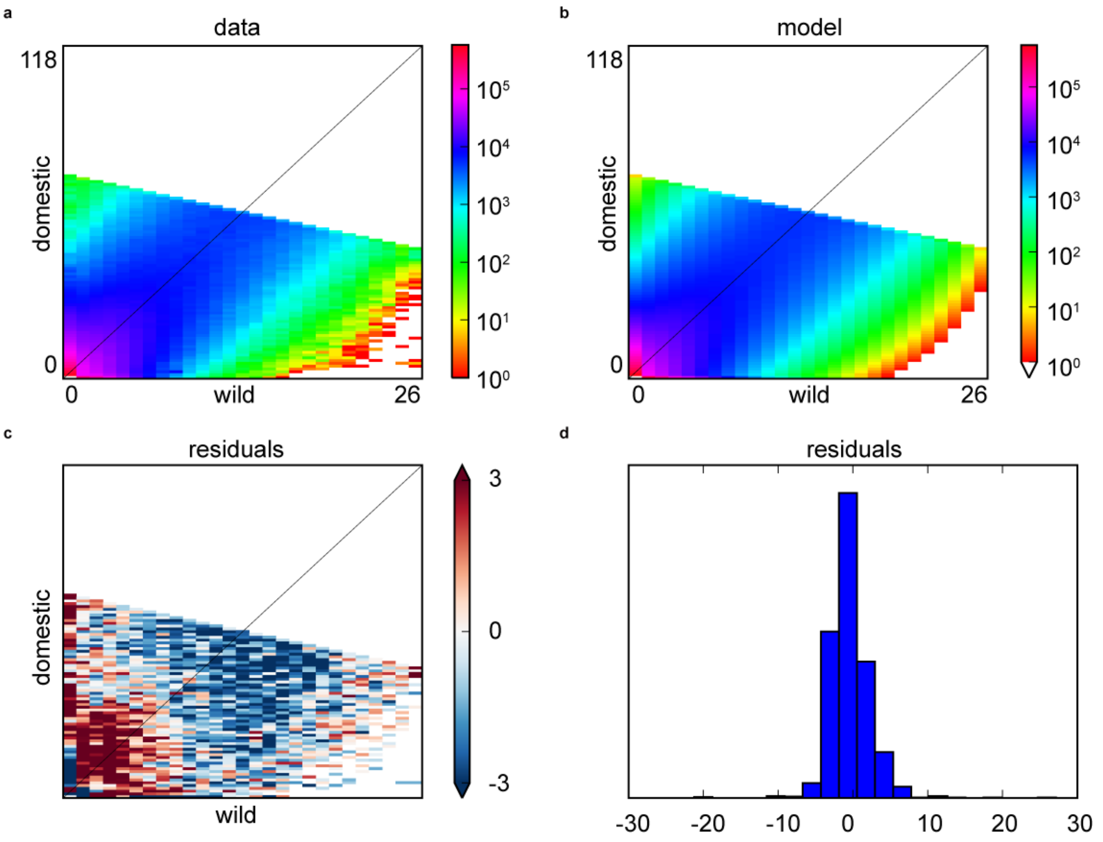

Supplementary Figure 7. Forward simulation and residuals analysis. The demographic parameters of the best model were used to perform forward simulation and residuals analysis with ∂a∂i.

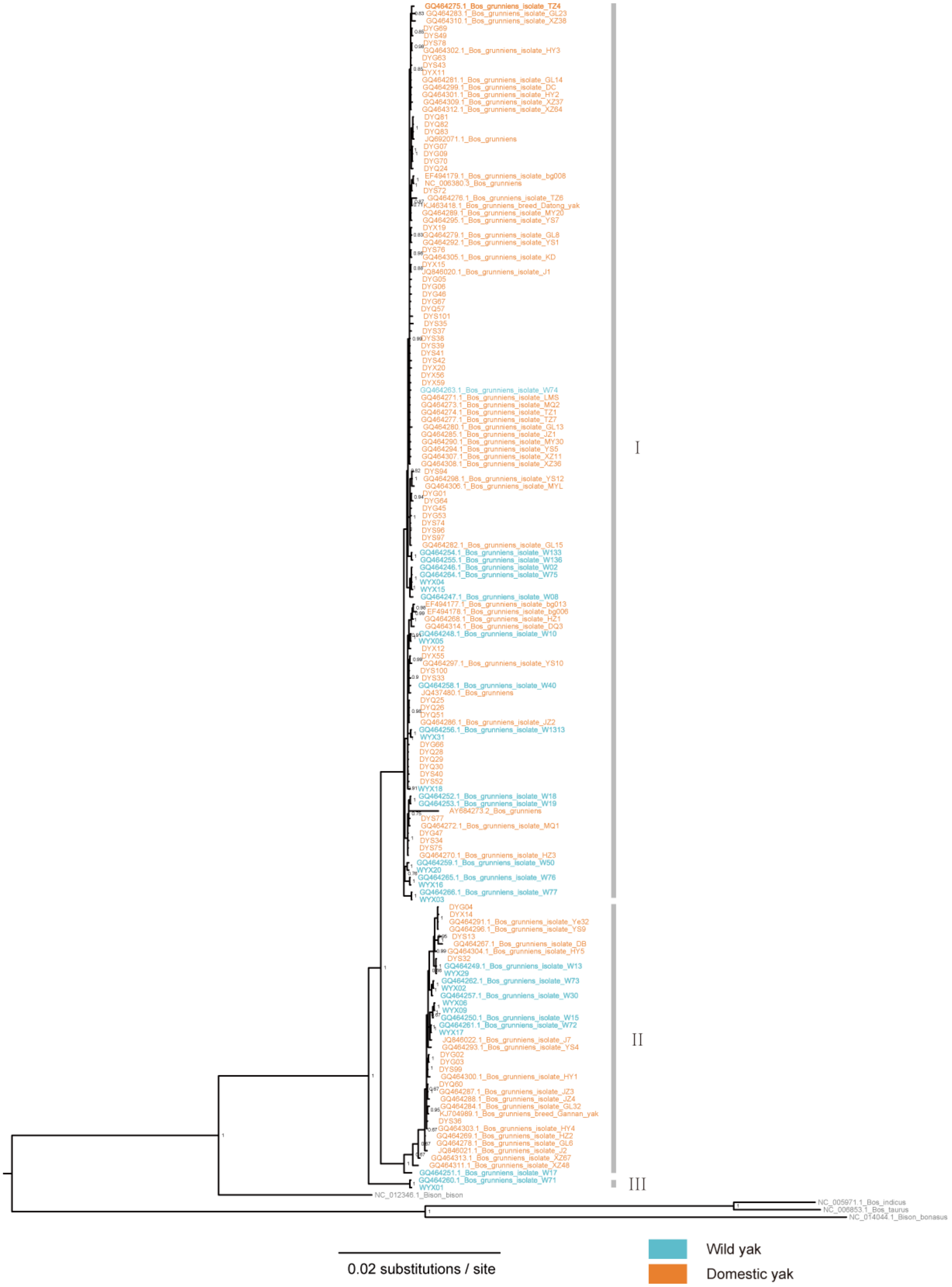

Supplementary Figure 8. Bayesian phylogenetic mtDNA tree. The labels of each external branch refer to the accession numbers of previously released mitochondrial genome sequences. Labels without an accession number refer to samples described in this study.

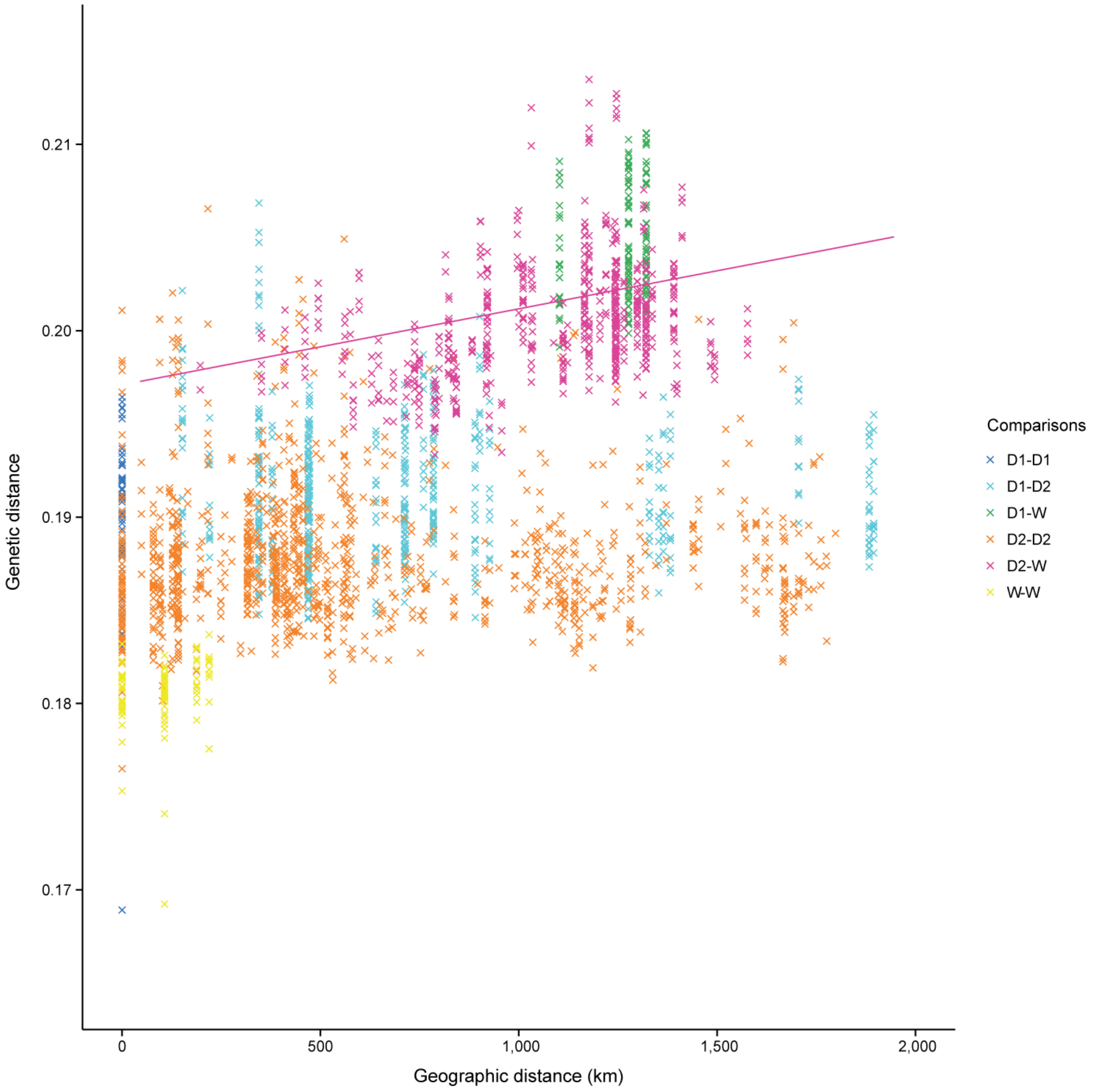

Supplementary Figure 9. Scatterplot of geographic distance and genetic distance. Comparisons within/between populations were represented by different colors. The regression line fitted to the data of D2-W ($D_{genetic}$ =0.196+(4.1×10^{-6}) $\times D_{geographic}$, R^2=0.128) is drawn in violet.

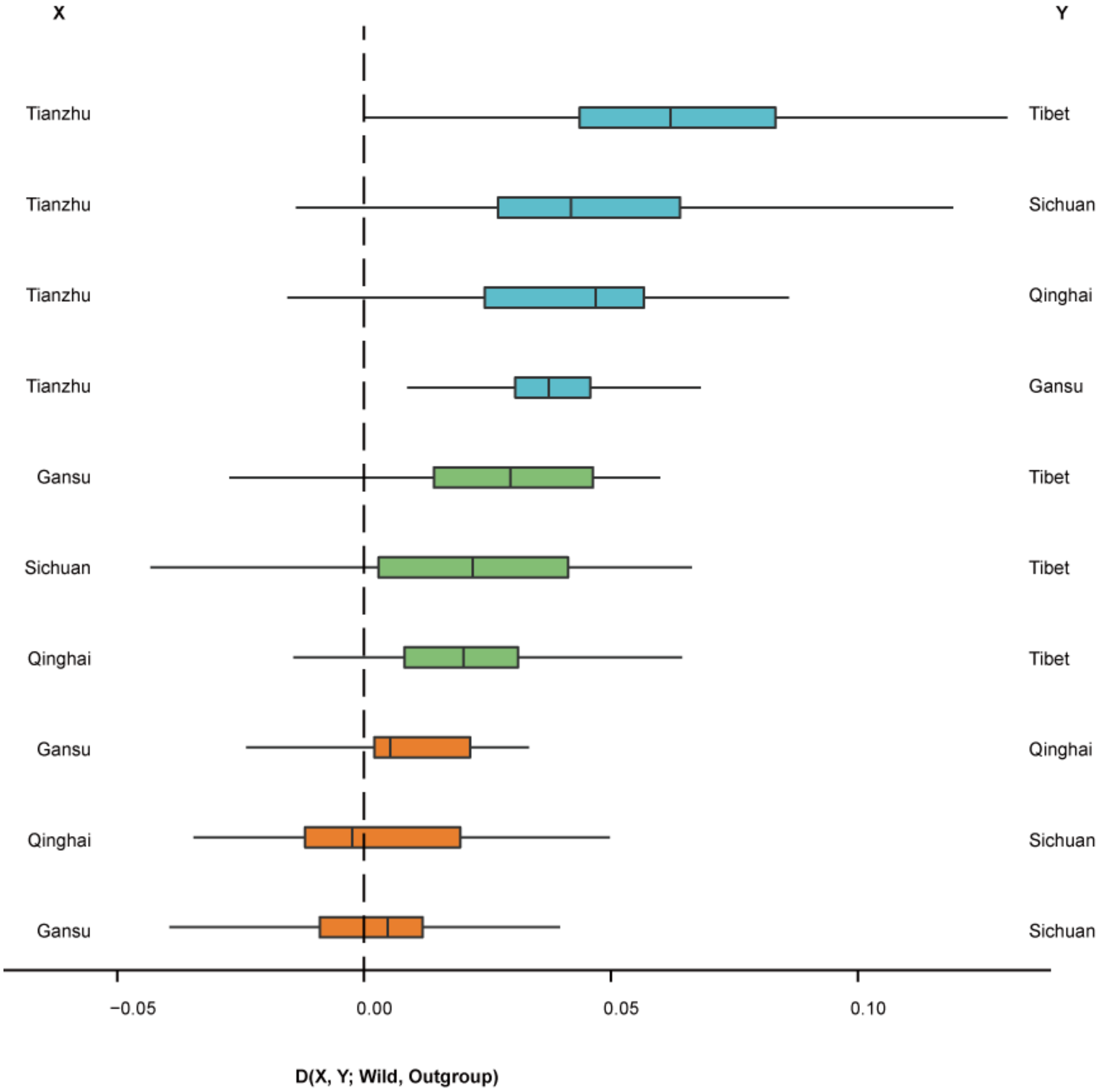

Supplementary Figure 10. Statistics testing whether the wild yak genome shares more derived alleles with one or the other of two domestic yak genomes (X, Y). We computed D statistics of the form D (X, Y, W, taurine cattle). A positive value indicates that Y is more related to wild yaks than X. The box in each box plot shows the lower quartile, and the median and upper quartile values.

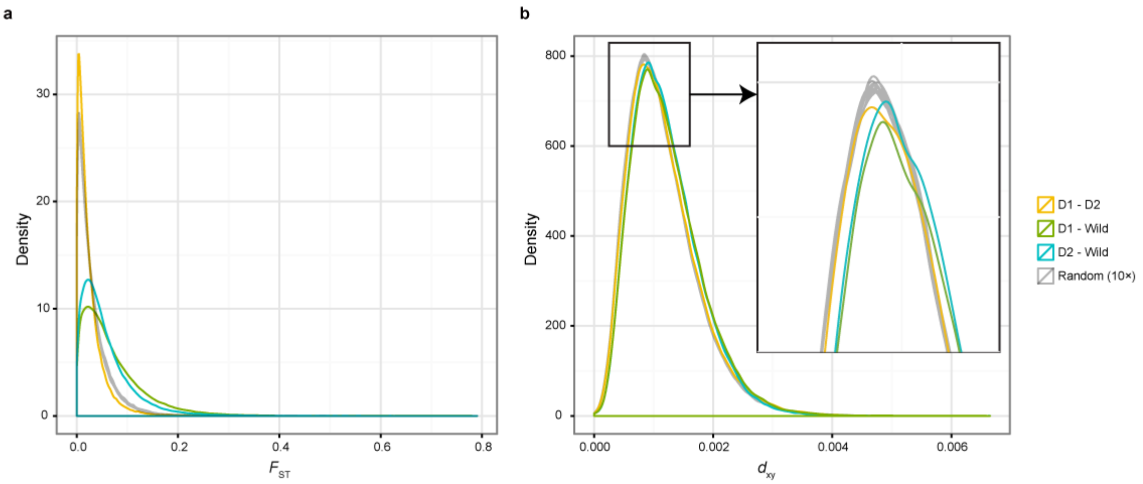

Supplementary Figure 11. Genome-wild distribution of F_{ST} (a) and d_{xy} (b). The grey lines represent the results from ten reshuffled combinations (R1 to R10).

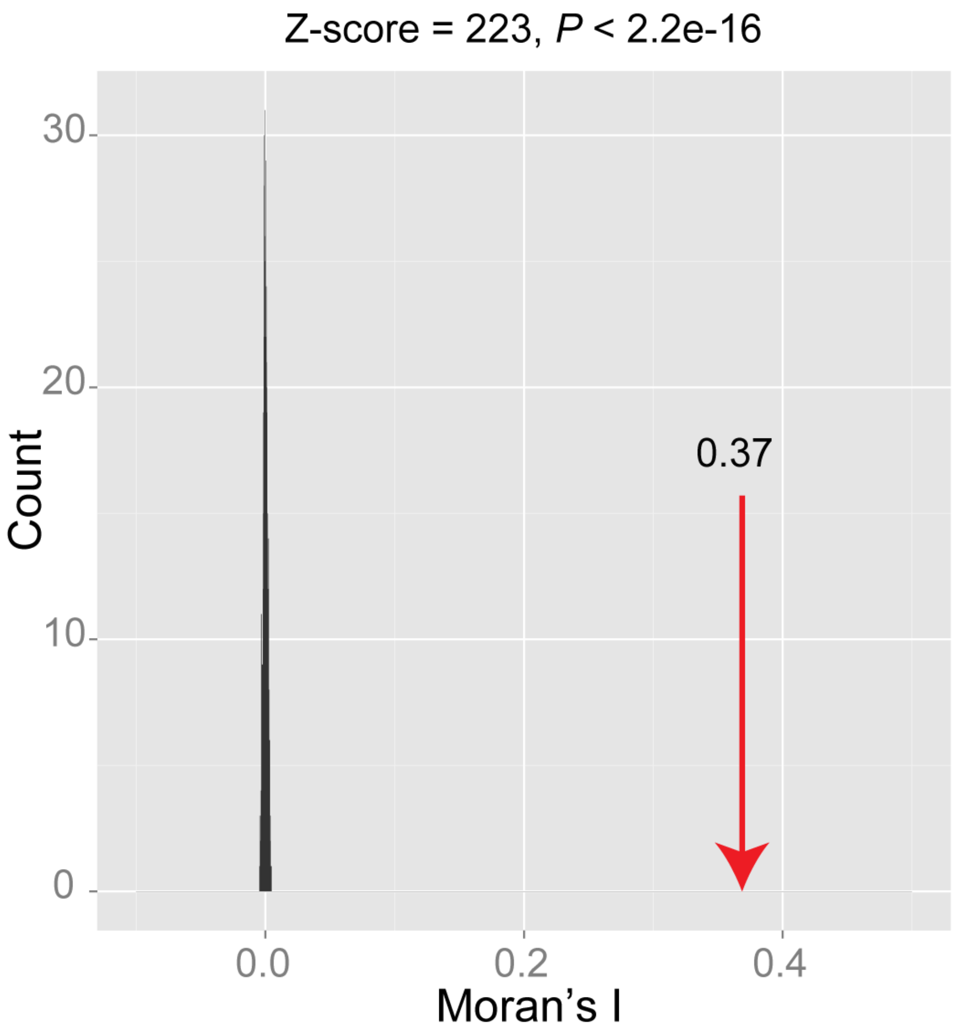

Supplementary Figure 12. Spatial autocorrelation statistics. Moran’s I was calculated to access the genome-wide clustering of the highly divergent (F_{ST}>0.2) regions within the genome. The histogram in black indicate Moran’s I calculated in reshuffled windows (1000 Monte Carlo simulations) while the red arrow indicates the real Moran's I.

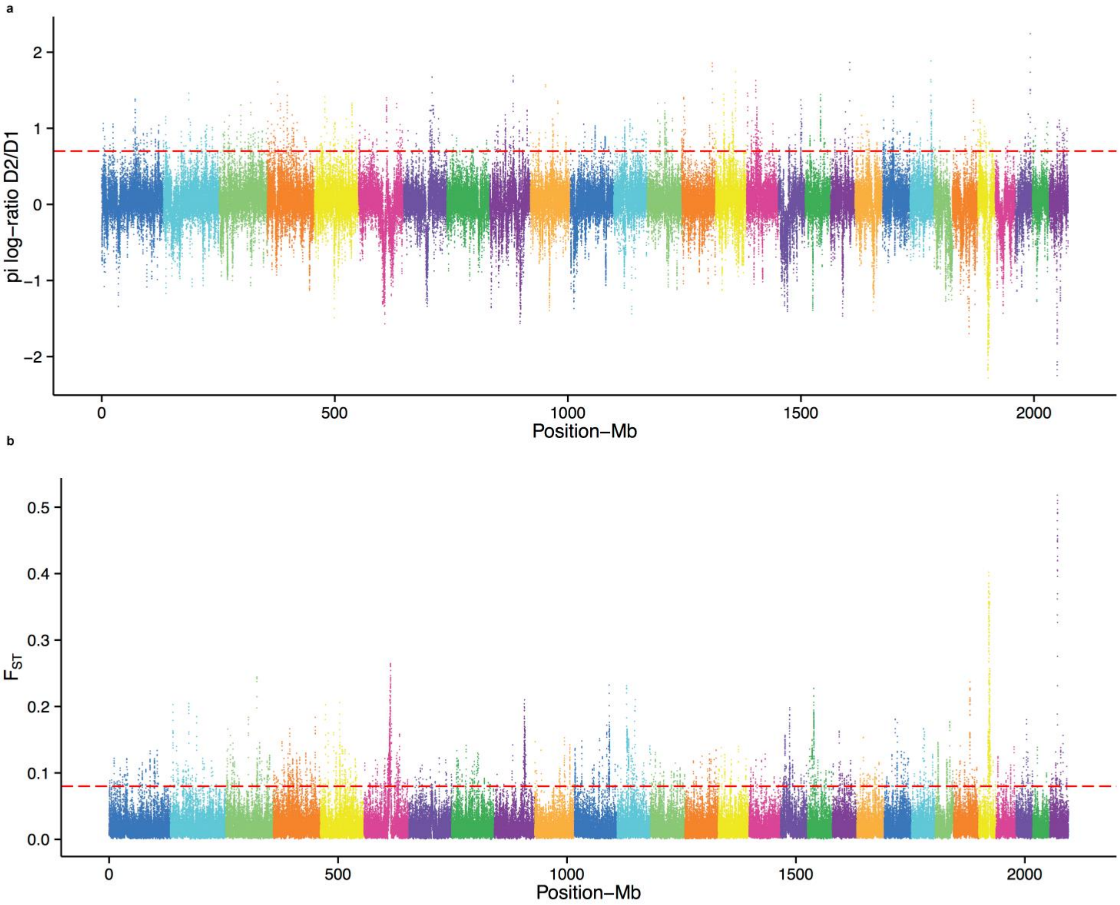

Supplementary Figure 13. Genome-wild distribution of ln ratio ($\theta_{\pi,D2}/\theta_{\pi,D1}$) (a) and F_{ST} (b) between D1 and D2 population. The horizontal dashed line indicates the threshold defining the improvement sweeps (ln ratio ≥0.70 and F_{ST} ≥0.08).

Supplementary Tables

1 **Supplementary Table 1. Overview of sample information and sequencing statistics.**

Sample ID	Description	Raw reads data (Gbp)	Raw Depth	Clean reads data (Gbp)	Map Ratio	Genome Coverage	Efeective Depth
DYG01	Domestic, Tianzhu Gansu, PRC	21.17	7.97	18.72	0.871	0.983	6.11
DYG02	Domestic, Tianzhu Gansu, PRC	21.48	8.08	19.14	0.886	0.981	6.35
DYG03	Domestic, Tianzhu Gansu, PRC	23.34	8.78	20.41	0.845	0.979	6.45
DYG04	Domestic, Tianzhu Gansu, PRC	22.79	8.58	19.63	0.922	0.981	6.85
DYG05	Domestic, Tianzhu Gansu, PRC	21.36	8.04	18.87	0.854	0.975	6.06
DYG06	Domestic, Tianzhu Gansu, PRC	21.94	8.26	19.00	0.869	0.977	6.25
DYG07	Domestic, Tianzhu Gansu, PRC	23.01	8.66	20.09	0.897	0.982	6.84
DYG09	Domestic, Tianzhu Gansu, PRC	21.30	8.02	18.75	0.916	0.982	6.47
DYG66	Domestic, Tianzhu Gansu, PRC	30.32	11.41	23.83	0.905	0.991	8.13
DYG69	Domestic, Tianzhu Gansu, PRC	21.58	8.12	18.72	0.936	0.983	6.72
DYG70	Domestic, Tianzhu Gansu, PRC	21.59	8.13	18.91	0.931	0.982	6.71
DYG47	Domestic, Hezuo Gansu, PRC	15.98	6.02	14.85	0.845	0.967	4.75
DYG45	Domestic, Maqu Gansu, PRC	20.46	7.70	17.83	0.929	0.983	6.31
DYG46	Domestic, Maqu Gansu, PRC	27.16	10.22	23.37	0.939	0.991	8.30
DYG53	Domestic, Maqu Gansu, PRC	19.97	7.52	19.69	0.893	0.987	6.67
DYG63	Domestic, Maqu Gansu, PRC	21.76	8.19	19.54	0.913	0.977	6.83
DYG64	Domestic, Maqu Gansu, PRC	20.43	7.69	16.95	0.932	0.979	6.04
DYG67	Domestic, Maqu Gansu, PRC	27.28	10.27	23.88	0.933	0.991	8.51
DYQ25	Domestic, Jianzha Qianghai, PRC	24.48	9.21	20.42	0.916	0.983	6.90
DYQ26†	Domestic, Jianzha Qianghai, PRC	22.78	8.57	18.92	0.919	0.979	6.44
DYQ51†	Domestic, Jianzha Qianghai, PRC	17.03	6.41	16.83	0.869	0.980	5.57
DYQ60	Domestic, Jianzha Qianghai, PRC	20.96	7.89	20.61	0.904	0.987	7.17
DYQ81†	Domestic, Nangqian Qinghai, PRC	25.32	9.53	21.20	0.923	0.988	7.50
DYQ82†	Domestic, Nangqian Qinghai, PRC	22.77	8.57	19.25	0.937	0.987	6.93

DYQ83†	Domestic, Nangqian Qinghai, PRC	27.22	10.25	23.27	0.938	0.992	8.38
DYQ28†	Domestic, Wudaoliang Qianghai, PRC	23.78	8.95	19.88	0.923	0.984	6.81
DYQ29†	Domestic, Wudaoliang Qianghai, PRC	22.51	8.47	19.15	0.926	0.983	6.58
DYQ30	Domestic, Wudaoliang Qianghai, PRC	28.01	10.54	23.22	0.910	0.989	7.75
DYQ24	Domestic, Yushu Qianghai, PRC	25.92	9.76	21.85	0.919	0.988	7.43
DYQ57	Domestic, Yushu Qianghai, PRC	19.21	7.23	18.25	0.881	0.983	6.15
DYS39	Domestic, Aba Sichuan, PRC	23.95	9.02	20.84	0.910	0.987	7.12
DYS42	Domestic, Aba Sichuan, PRC	26.03	9.80	23.26	0.942	0.990	8.41
DYS43	Domestic, Aba Sichuan, PRC	23.64	8.90	20.82	0.932	0.985	7.41
DYS44*	Domestic, Aba Sichuan, PRC	28.90	10.88	23.63	0.890	0.931	7.67
DYS72	Domestic, Aba Sichuan, PRC	21.26	8.00	17.39	0.915	0.978	6.06
DYS13	Domestic, Dege Sichuan, PRC	21.86	8.23	19.50	0.925	0.984	6.71
DYS32	Domestic, Dege Sichuan, PRC	22.27	8.38	18.88	0.923	0.980	6.51
DYS33	Domestic, Dege Sichuan, PRC	27.84	10.48	22.71	0.912	0.987	7.73
DYS75	Domestic, Dege Sichuan, PRC	25.12	9.45	21.54	0.919	0.987	7.52
DYS34	Domestic, Hongyuan Sichuan, PRC	21.60	8.13	18.27	0.937	0.984	6.60
DYS35	Domestic, Hongyuan Sichuan, PRC	21.77	8.19	18.53	0.948	0.985	6.66
DYS37	Domestic, Hongyuan Sichuan, PRC	26.98	10.16	22.91	0.911	0.989	7.64
DYS38	Domestic, Hongyuan Sichuan, PRC	29.10	10.95	24.62	0.941	0.992	8.83
DYS40	Domestic, Hongyuan Sichuan, PRC	21.56	8.12	17.64	0.937	0.980	6.20
DYS41	Domestic, Hongyuan Sichuan, PRC	21.38	8.05	18.71	0.929	0.984	6.60
DYS49	Domestic, Hongyuan Sichuan, PRC	20.48	7.71	20.21	0.905	0.988	7.01
DYS78	Domestic, Hongyuan Sichuan, PRC	25.41	9.57	21.45	0.906	0.982	7.37
DYS94	Domestic, Jiulong Sichuan, PRC	15.16	5.71	14.92	0.886	0.969	4.90
DYS96	Domestic, Jiulong Sichuan, PRC	15.16	5.70	15.05	0.928	0.977	5.43
DYS97	Domestic, Jiulong Sichuan, PRC	15.25	5.74	15.05	0.908	0.976	5.27
DYS100	Domestic, Kangding Sichuan, PRC	15.32	5.76	15.14	0.900	0.969	5.16
DYS101	Domestic, Kangding Sichuan, PRC	15.29	5.76	15.12	0.931	0.973	5.26
DYS76	Domestic, Kangding Sichuan, PRC	22.61	8.51	19.65	0.899	0.980	6.73
DYS99	Domestic, Kangding Sichuan, PRC	15.26	5.74	15.05	0.907	0.971	5.18

DYS36	Domestic, Ruoergai Sichuan, PRC	20.92	7.87	17.79	0.930	0.981	6.37
DYS52	Domestic, Ruoergai Sichuan, PRC	17.41	6.55	17.10	0.896	0.983	5.87
DYX55	Domestic, Dangxiong Tibet, PRC	21.79	8.20	21.43	0.895	0.987	7.32
DYX14	Domestic, Jiangda Tibet, PRC	22.23	8.37	19.83	0.930	0.984	6.89
DYX15	Domestic, Jilong Tibet, PRC	21.64	8.14	19.63	0.932	0.986	6.94
DYX59	Domestic, Lhasa Tibet, PRC	17.76	6.68	17.46	0.863	0.980	5.69
DYX56	Domestic, Namucuo Tibet, PRC	16.12	6.07	15.84	0.886	0.977	5.35
DYX19	Domestic, Nielamu Tibet, PRC	21.77	8.19	19.45	0.919	0.981	6.62
DYX20	Domestic, Nielamu Tibet, PRC	22.60	8.51	20.28	0.916	0.982	6.89
DYX48*	Domestic, Nielamu Tibet, PRC	15.48	5.83	14.35	0.837	0.961	4.49
DYS74	Domestic, Shiqu Sichuan, PRC	21.90	8.24	19.58	0.938	0.984	7.05
DYS77	Domestic, Shiqu Sichuan, PRC	23.09	8.69	20.28	0.925	0.987	7.16
DYX12	Domestic, Yadong Tibet, PRC	22.23	8.37	19.68	0.926	0.983	6.81
DYX11	Domestic, Zedang Tibet, PRC	22.69	8.54	20.33	0.927	0.985	7.07
DYY31*	Domestic, Deqin Yunnan, PRC	25.40	9.56	20.85	0.921	0.982	7.20
WYX01	Wild, Kokohili region	16.65	6.27	15.59	0.804	0.962	4.71
WYX02	Wild, Kokohili region	17.95	6.76	16.40	0.846	0.967	5.08
WYX03	Wild, Kokohili region	16.39	6.17	15.24	0.833	0.962	4.74
WYX04	Wild, Kokohili region	17.11	6.44	16.77	0.880	0.980	5.61
WYX05	Wild, Kokohili region	23.11	8.70	22.82	0.850	0.988	7.37
WYX06†	Wild, Kokohili region	23.62	8.89	23.31	0.885	0.990	7.87
WYX09	Wild, Kokohili region	25.59	9.63	25.21	0.902	0.990	8.64
WYX15	Wild, Kokohili region	22.17	8.34	21.84	0.895	0.988	7.46
WYX16	Wild, Kokohili region	19.63	7.39	19.38	0.877	0.985	6.45
WYX17	Wild, Kokohili region	23.19	8.73	22.86	0.880	0.989	7.64
WYX18	Wild, Kokohili region	24.85	9.35	21.77	0.868	0.984	6.90
WYX20	Wild, Kokohili region	22.33	8.41	19.55	0.932	0.985	6.94
WYX29	Wild, Kokohili region	36.41	13.71	24.26	0.539	0.927	4.71
WYX31	Wild, Kokohili region	29.81	11.22	25.47	0.716	0.977	6.80
WYX27*	Wild, Kokohili region	23.45	8.82	20.22	0.727	0.931	4.95

* samples that have been discarded because of high missing percentage (>50%).

† samples that have been discarded because of high pairwise genetic similarity (IBS>0.9).

Supplementary Table 2. Distribution of SNPs within various yak genomic regions.

Variant type	SNP Count
intergenic region	11,116,198
intron variant	3,251,596
downstream gene variant	549,423
upstream gene variant	538,512
synonymous variant	62,828
missense variant	51,502
splice region variant	8,627
stop gained	1,029
splice donor variant	220
splice acceptor variant	155
start lost	66
stop lost	39
stop retained variant	39
initiator codon variant	3

Supplementary Table 3. Tracy-Widom (*TW*) statistics and *P*-values for the ten first eigenvalues in PCA. The significant p-values are in bold.

Number	Eigenvalues	*TW*	*P*
1	3.24	70.085	**6.48E-172**
2	1.36	27.81	**2.12E-44**
3	1.28	23.295	**1.80E-34**
4	1.17	8.307	**9.72E-09**
5	1.14	6.489	**5.43E-07**
6	1.11	2.152	**0.00802954**
7	1.11	3.293	**0.000959434**
8	1.08	-1.026	0.4241
9	1.06	-3.994	0.992308
10	1.06	-4.646	0.998971

Supplementary Table 4. Population genetic summary statistics.

**Population**	θ_π	θ_w	**Tajima's *D***	F_{ST}	
				D2	W
D1	0.00137	0.00134	0.137	0.0213	0.0742
D2	0.00138	0.00138	0.053	-	0.0585
D1+D2	0.00140	0.00147	-0.100	-	0.0582
W	0.00131	0.00121	0.224	-	-

Supplementary Table 5. GO analysis of positively selected genes.

GO term	Function	Adjusted p-value	Gene count*
GO:0051969	regulation of transmission of nerve impulse	3.02E-02	8(249)
GO:0048167	regulation of synaptic plasticity	1.77E-02	5(98)
GO:0050808	synapse organization	4.01E-02	4(75)
GO:0051928	positive regulation of calcium ion transport	2.77E-03	4(48)
GO:0010676	positive regulation of cellular carbohydrate metabolic process	3.09E-02	3(40)
GO:0014068	positive regulation of phosphatidylinositol 3-kinase cascade	1.90E-02	3(37)
GO:0001919	regulation of receptor recycling	1.87E-02	2(14)
GO:0014047	glutamate secretion	1.87E-02	2(14)
GO:0045005	maintenance of fidelity involved in DNA-dependent DNA replication	1.77E-02	2(12)
GO:0048488	synaptic vesicle endocytosis	1.77E-02	2(13)
GO:2000811	negative regulation of anoikis	1.77E-02	2(13)
GO:0043649	dicarboxylic acid catabolic process	1.07E-02	2(11)
GO:0006379	mRNA cleavage	5.03E-03	2(10)
GO:0010919	regulation of inositol phosphate biosynthetic process	5.03E-03	2(10)
GO:0090102	cochlea development	5.03E-03	2(10)
GO:0001921	positive regulation of receptor recycling	8.56E-04	2(8)
GO:0060732	positive regulation of inositol phosphate biosynthetic process	8.56E-04	2(8)
GO:0031297	replication fork processing	4.67E-04	2(7)
GO:0044430	cytoskeletal part	3.29E-02	24(1307)
GO:0005882	intermediate filament	1.92E-05	8(127)
GO:0045095	keratin filament	1.03E-04	5(56)
GO:0033268	node of Ranvier	1.77E-02	2(13)
GO:0005662	DNA replication factor A complex	8.56E-04	2(8)
GO:0035748	myelin sheath abaxonal region	8.56E-04	2(8)
GO:0016528	sarcoplasm	1.92E-05	2(5)
GO:0016646	oxidoreductase activity, acting on the CH-NH group of donors, NAD or NADP as acceptor	3.77E-02	2(16)
GO:0016755	transferase activity, transferring amino-acyl groups	3.77E-02	2(16)
GO:0032794	GTPase activating protein binding	8.56E-04	2(8)

*For each term, gene count shows number of genes in selective sweeps relative to total number of annotated genes (in parentheses).

Supplementary Table 6. Putative regions identified to be under improvement sweeps

Chr*	Scaffold	Regions	Gene ID	Description	
1	scaffold374_1	1,660,000-1,780,000	ENSP00000229268-D2	USP13	Ubiquitin carboxyl-terminal hydrolase 13
			ENSP00000419975-D1	PEX5L	PEX5-related protein
4	scaffold2014_1	300,000-400,000	-	-	-
11	scaffold1630_1	250,000-490,000	-	-	-
18	scaffold473_1	60,000-350,000	ENSBTAP00000004555-D1	ZNF821	Zinc finger protein 821
			ENSBTAP00000019734-D1	MYLK3	Putative myosin light chain kinase 3
			ENSBTAP00000030826-D25	-	-
			ENSP00000219097-D1	ORC6L	Origin recognition complex subunit 6
			ENSP00000299138-D1	VPS35	Vacuolar protein sorting-associated protein 35
			ENSBTAP00000044758-D1	SHCBP1	SHC SH2 domain-binding protein 1
		450,000-650,000	ENSP00000268699-D1	GAS8	Growth arrest-specific protein 8
			ENSBTAP00000027077-D1	DBNDD1	Dysbindin domain-containing protein 1
			ENSBTAP00000046304-D9	Afg3l1	AFG3-like protein 1
			ENSP00000393854-D1	CENPBD1	CENPB DNA-binding domain-containing protein 1
			ENSP00000268676-D1	DEF8	Differentially expressed in FDCP 8 homolog
			ENSP00000302777-D1	TUBB3	Tubulin beta-3 chain
			ENSBTAG00000023731-D1	MC1R	Melanocortin 1 Receptor
			ENSP00000263346-D1	Tcf25	Transcription factor 25
			ENSBTAP00000001541-D1	SPIRE2	Protein spire homolog 2
			ENSBTAP00000002478-D1	FANCA	Fanconi anemia group A protein
			ENSP00000415836-D1	ZNF276	Zinc finger protein 276
			ENSBTAP00000028205-D1	Rapgef5	Rap guanine nucleotide exchange factor 5
21	scaffold3174_1	550,000-590,000	-	-	-
24	scaffold1008_1	200,000-250,000	-	-	-
25	scaffold5_2	680,000-700,000	ENSBTAP00000016726-D1	TEKT4	Tektin-4

			ENSP00000414871-D1	LOC442028	HCG1820743, isoform CRA_a
UN	scaffold3256_1	20,000-50,000	-	-	-

* Putative chromosome positions was identified by whole-genome alignment of the yak and cattle genomes using Blastz. UN represents no hit was found in cattle genome.

Supplementary Notes

Supplementary Note 1. Population structure and gene flow

The NGSadmix[1] analysis revealed a similar population structure to that observed in the NJ trees and PCA analysis. Wild and domestic yaks were clearly distinguished when the model was fitted using two ancestral components (K=2). K=3 differentiated D1 and D2 yaks. Genetic distances of D2 individuals with wild animals increase with the corresponding geographic distance, but the D2 population shows no other evidence of geographic population structuring and within the D2 populations there is no correlation of geographic and genetic inter individual distance confirming previous analyses[2,3] (Supplementary Fig. 9). This may reflect gene flow between wild and domestic yaks and gene flow between breeds after commercial trade.

To investigate the gene flow between the two populations of domestic yaks (D1 and D2) and the wild yaks (W), we conducted the D-statistic[4] for three quartets types: (D1, D2; W, Outgroup), and (D1, D1; W, Outgroup), (D2, D2; W, Outgroup). The cattle genome was used as outgroup. This statistic analysis revealed a statistically significant excess of shared derived alleles between the D2 and wild yaks in the (D1, D2; W, Outgroup) quartet (Supplementary Fig. 10 and Supplementary Data 3 for details). Symmetry tests indicated that wild yaks are more closely related to D2 than to D1 yaks, most likely reflecting genetic drift in the D1 population by intensive breeding. This result demonstrates the profound and rapid impact of human breeding on domestic yaks, which has taken place over a relatively short period (~130 years)[5]. Most of the (D2, D2; W, Outgroup) quartet yielded a statistically significant excess of shared alleles between D2 and wild yaks. In agreement with Supplementary Figure 9, the strongest evidence of gene

flow between the D2 and wild yaks was found for yaks from the Tibet autonomous region.

The best coalescent simulation model inferred by fastsimcoal2 also indicates a continuous and extensive bidirectional gene flow between domestic and wild yaks, which is compatible with the D-statistics (Supplementary Data 3) and the low level of differentiation between populations (Fig. 1b and Supplementary Table 4). This is not surprising given the breeding practices. Many domestic yaks are kept in the same environment as wild yaks and allowed to roam in a semi-managed state, where the large wild bulls outcompete the domestic bulls. Herdsmen even prefer offspring of wild-domestic crosses and frequently drive receptive domestic yaks into regions inhabited by wild yaks. Conversely, domestic populations influence wild populations if local Tibetan communities set domestic yaks free to thank their environment for sustaining them[5].

Supplementary Note 2. Estimation of linkage disequilibrium and inbreeding.

We compared patterns of linkage disequilibrium (LD) among domestic and wild populations. LD decay was calculated on the basis of the r^2 values and the corresponding distances between paired SNPs. Pairwise r^2 values were calculated for SNPs with minor allele frequencies (MAF) greater than 0.05 using Haploview software[6] with the parameters '-dprime -maxDistance 1000 -minMAF 0.05 -hwcutoff 0.001 -missingCutoff 0.5 -minGeno 0.6'. To minimize bias due to sample size variation, we randomly reduced the size of the D2 and W yak data set to the size of the D1 yak dataset (11 samples). The LD level was higher for domestic populations (D1 and D2) than for the wild population,

with r2 dropping below 0.3 at about 18 kb, 14.5 kb and 5.5 kb in D1, D2 and wild populations, respectively (Supplementary Fig. 1e).

As LD patterns can be influenced by non-random mating, we also estimated inbreeding coefficients (F) using ngsF[7] under a probabilistic framework that take accounts for the uncertainty of each genotype's assignation. We found that the inbreeding level is about three times higher in domestic populations (F_{D1}=5.32%, F_{D2}=6.37%) than in the wild population (F_W=1.72%), which may explain the higher LD in domestic populations. Inbreeding leading to genetic subdivision may also explain the large spread in the inter individual genetic distances (Supplementary Fig. 9).

Supplementary Note 3. Divergence in Tianzhu white yaks

As in breeds of most other domestic species, coat color is the obvious target of selection. The Tianzhu white yak (D1) derived from D2 population is for yaks the only typical modern improved breed, which since the late 19th century has been developed within the D2 population by intensive artificial selection with standardized criteria for the white coat color and for a high fiber production[5]. In the D2 only 2-3 percent of the animals are white. Because of traditional mobile livestock raising practice and an absence of parentage records, the mode of inheritance patterns and the genetic basis of white color of yak remain unknown.

To investigate the genetic differentiation between D1 and D2 populations, we performed whole-genome scan of F_{ST} and d_{xy} (mean pairwise nucleotide difference) between D1 and D2 with 50 kb windows and 10 kb steps. In addition, we performed 10 scans (R1 to R10) of reshuffled sample combination of D1 and D2. The F_{ST} distribution

of the D1-D2 comparison and of R1 to R10 were similar and were for D1-D2 not significantly larger than for R1 to R10 (P>0.68, Kolmogorov-Smirnov test, Supplementary Fig. 11a). Distributions of d_{xy} distribution were also similar, although d_{xy} is larger in D1-D2 ($P<6.26\times10^{-13}$, Supplementary Fig. 11b). Thus, for most part of the genome the D1-D2 genetic distance is close to the distance in random selected populations and is much smaller than the corresponding distances between domestic and wild yak. However, the most diverged windows (F_{ST} between D1 and D2 >0.2), covering about 3.28 Mb, tend to aggregate in 23 scaffolds, which is significant according to spatial autocorrelation analysis (Supplementary Fig. 12). Considering the recent breeding history, this most likely reflecting selective sweeps.

In order to identify the improvement sweeps, we scanned genomic regions with a drastic reduction in nucleotide diversity of D1 and extreme divergence in allele frequency in D1 and D2 populations (π log-ratio D2/D1 and F_{ST}, Supplementary Fig. 13). In total, we identified 9 improvement sweeps with the highest signal (P<0.005, Z test) covering 1.09 Mb (0.04%) of the genome. Only 4 of the 9 sweep regions harbored coding genes and we identified a total of 22 genes (Supplementary Table 6). Among these, two genes are related to pigment synthesis: *USP13* and *MC1R*. *USP13* specifically mediates deubiquitination of target protein *MITF*, a master regulator of melanocyte development[8,9]. *MC1R* encodes the receptor protein for melanocyte-stimulating hormone, an regulator of melanin production. It is a well-known coat color gene in a range of species[9,10] and is in yak incorporated in the longest sweep region. Eighteen genes within 427 kb from *USP13* or *MC1R* are likely to be involved in the selective sweeps hitch-hiking. The results are consistent with the differences in coat color observed for D1 and D2 yaks. Two genes

incorporated in scaffold5_2 and potential regular elements located in the rest five sweep regions without gene annotation may affect other D1-D2 differences with no clear morphological traits.

Overall, we note that the D1 population is not only genetically close to D2 population but also underwent a limited number of selective sweeps partially associated with coat color during its breeding.

Supplementary References

1. Skotte, L., Korneliussen, T.S. & Albrechtsen, A. Estimating individual admixture proportions from next generation sequencing data. *Genetics* **195**, 693-702 (2013).
2. Guo, S. *et al.* Origin of mitochondrial DNA diversity of domestic yaks. *BMC Evol. Biol.* **6**, 73 (2006).
3. Wang, Z. F. *et al.* Phylogeographical analyses of domestic and wild yaks based on mitochondrial DNA: new data and reappraisal. *J. Biogeogr.* **37**, 2332-2344 (2010).
4. Durand, E.Y., Patterson, N., Reich, D. & Slatkin, M. Testing for ancient admixture between closely related populations. *Mol. Biol. Evol.* **28**, 2239-52 (2011).
5. Wiener, G., Jianlin, H. & Ruijun, L. *The yak*, (FAO Regional Office for Asia and the Pacific Food and Agriculture Organization of the United Nations, Bangkok, Thailand, 2003).
6. Barrett, J. C. Haploview: Visualization and analysis of SNP genotype data. *Cold Spring Harb. Protoc.* **2009**, pdb ip71 (2009).
7. Vieira, F.G., Fumagalli, M., Albrechtsen, A. & Nielsen, R. Estimating inbreeding coefficients from NGS data: Impact on genotype calling and allele frequency estimation. *Genome Res.* **23**, 1852-61 (2013).
8. Levy, C., Khaled, M. & Fisher, D.E. MITF: master regulator of melanocyte development and melanoma oncogene. *Trends Mol. Med.* **12**, 406-14 (2006).
9. Linderholm, A. & Larson, G. The role of humans in facilitating and sustaining coat colour variation in domestic animals. *Semin. Cell Dev. Biol.* **24**, 587-93 (2013).
10. Switonski, M., Mankowska, M. & Salamon, S. Family of melanocortin receptor (MCR) genes in mammals-mutations, polymorphisms and phenotypic effects. *J. Appl. Genet.* **54**, 461-72 (2013).
